# Effects of *Portulaca Oleracea* Extract on Acute Alcoholic Liver Injury of Rats

**DOI:** 10.3390/molecules24162887

**Published:** 2019-08-08

**Authors:** Jing-Yi Qiao, Han-Wei Li, Fu-Gang Liu, Yu-Cheng Li, Shuo Tian, Li-Hua Cao, Kai Hu, Xiang-Xiang Wu, Ming-San Miao

**Affiliations:** 1 Scientific Research and Experiment Center, Henan University of Chinese Medicine, Zhengzhou 450046, China; 2 Department of Medicine, Henan University of Chinese Medicine, Zhengzhou 450046, China; 3 Graduate School, Henan University of Chinese Medicine, Zhengzhou 450046, China

**Keywords:** *Portulaca oleracea extract* (POE), acute alcoholic liver injury, oxidative stress, inflammatory reaction, lipid metabolism, miR-122

## Abstract

The present study was envisaged to investigate the chemical constituents and the intervention effects of *Portulaca oleracea* extract (POE) on acute alcoholic liver injury of rats. The chemical composition of POE was detected by high performance liquid chromatography (HPLC). Sixty male Wistar rats were divided into 6 groups: Normal control (NC) group, acute alcoholic liver injury model group (ALI), low, medium and high dose of POE (25, 50, 100 mg/kg) groups and bifendate (BF, 3.75 mg/kg) group. Each group was given by intragastrical administration for 7 days. Alcoholic liver injury was induced in the experimental model by administering 50% ethanol at 8 mL/kg and repeated administration after 6 h, for a period of 7 days. The results showed that pretreatment with POE significantly reduced the ethanol-elevated serum level of alanine aminotransferase (ALT), aspartate aminotransferase (AST), alkaline phosphatase (ALP) and triglyceride (TG). The activities of superoxide dismutase (SOD) and glutathione peroxidase (GSH-PX) in liver were enhanced followed by administration of POE, while the content of nitric oxide (NO) and malondialdehyde (MDA) was found to decrease. Hepatic content of tumor necrosis factor-α (TNF-α), and interleukin-6 (IL-6) was also reduced by POE treatment. These results indicated that POE could increase the antioxidant capacity and relieve the inflammatory injury of the liver cells induced by ethanol. Meanwhile, in our study, POE reduced the expression of miR-122, acetyl coenzyme A carboxylase (ACC) 1 mRNA and protein and increased the expression of lipoprotein lipase (LPL) mRNA and protein in liver, which indicated that POE could improve the lipid metabolism disorder induced by ethanol. Our findings suggested that POE had protective effects on acute alcoholic liver injury of rats.

## 1. Introduction

Alcoholic liver disease (ALD) is a liver metabolic disorder that is caused by acute or chronic alcohol consumption [1]. With the increase of unhealthy dietary habits, alcoholism and alcohol dependence, the incidence of ALD has increased significantly and become the second largest liver disorder after viral hepatitis, which seriously harms human health. It is reported that excessive drinking causes about 3 million deaths each year, accounting for 5.3% of all deaths in the world according to the World Health Organization (WHO) global status report on alcohol and health [2]. ALD mainly includes steatosis, hepatitis, fibrosis and cirrhosis. Fatty liver (i.e., steatosis) is the earliest response to heavy drinking and is characterized by the deposition of fat in hepatocytes. Under continued drinking, steatosis can proceed to steatohepatitis, fibrosis, cirrhosis and even liver cancer [3].

The pathogenesis of ALD is related to the toxicity of ethanol and its metabolites to liver, oxidative stress, lipid peroxidation, inflammatory cytokines, immunity, dysbiosis and environmental and individual factors [4,5,6,7,8]. Ethanol is mainly metabolized by alcohol dehydrogenase (ADH) and the microsome ethanol oxidation system (MEOS) into acetaldehyde, which is responsible for the generation of reactive oxygen species (ROS). The accumulation of ROS by ethanol metabolism causes the oxidative stress, which further leads to a chain reaction of lipid peroxidation, endoplasmic reticulum stress and inflammation caused by liver damage [9,10,11]. Acute alcohol consumption results in ALD, associated with an increase in ethanol-induced CYP2E1 activity, which can induce the oxidative stress and lipid peroxidation [12,13]. Excessive alcohol intake would also cause obstacles in the circulation of tricarboxylic acid and the oxidation of fatty acids and abnormal accumulation of lipids in liver, which can negatively affect the lipid metabolism [14,15].

MiR-122 is a kind of microRNA with specific and high expression in liver, which is involved in the process of various liver diseases and is highly expressed in models of various liver injuries. The role of miR-122 as a promising blood-based biomarker of the liver injury has been verified in alcoholic and inflammatory liver diseases [16,17]. MiR-122 has been studied for its participation in the regulation of lipid metabolism. MiR-122 inhibition was reported to regulate the gene expression involved in fatty acid synthesis and oxidation, which reduced the plasma cholesterol and triglyceride levels and significantly improved the liver steatosis [18,19]. Furthermore, the accumulation of lipids can promote the excessive expression of a large number of inflammatory cytokines like the tumor-necrosis factor (TNF) and other inflammatory factors [20]. Given the regulation of miR-122 in the lipid metabolism and its close relation to oxidation and inflammation, it has an important clinical value while developing potential drugs that could prevent and early cure alcoholic liver injury in order to reduce the incidence of ALD.

The dry aerial part of *Portulaca oleracea* L. belongimg to the *Portulacaceae* family, is widely distributed in many parts of the world, which is a kind of medicinal and edible medicine. It contains a diverse phyto-constituents that mainly includes the flavonoids, alkaloids, terpenoids, polysaccharides and organic acid and other classes of natural ingredients including fatty acids, vitamins, sterols, proteins, and minerals, which are essential for human health [21]. Flavonoids are one of the most effective chemical constituents of *Portulaca oleracea*, which mainly include quercetin, myricetin, luteolin, kaempferol, apigenin, genistein, and genistin [22]. Certain new flavonoids were isolated, such as oleracone C (1), D (2) and E (3) [23]. *Portulaca oleracea* has a wide range of pharmacological properties, such as antioxidant, analgesic, anti-inflammatory, neuroprotective, hepatoprotective, antitumor and immuno-modulation [24,25,26,27,28,29,30]. It has been used to be treatment various diseases including stomach illnesses, liver inflammation, respiratory diseases, fever, headache, wounds, ulcers, etc. [31]. Furthermore, *Portulaca oleracea* is known to regulate the sugar and lipid metabolism in the body [32,33]. Bifendate is frequently selected as a positive control drug for the evaluating potential hepatoprotective agents, which can play a major therapeutic role in the regulation of immunity and inflammation for acute liver injury [34,35,36]. Hence, we selected bifendate as a positive control drug for evaluating the hepatoprotective effects of *Portulaca oleracea* extract (POE).

Currently, there is no systematic report on the chemical constituents and hepatoprotective effects of POE on acute alcoholic liver injury. Therefore, this study focused on estimating the main components of POE and the intervention effects of POE on acute alcoholic liver injury of rats. Additionally, we investigated the effects of POE on the expression of miR-122 and its related genes and protein and explored the underlying mechanism of POE on acute alcoholic liver injury, which can provide an experimental basis for its clinical application.

## 2. Results

### 2.1. Analysis of Chemical Constituents of POE

**Figure 1:**
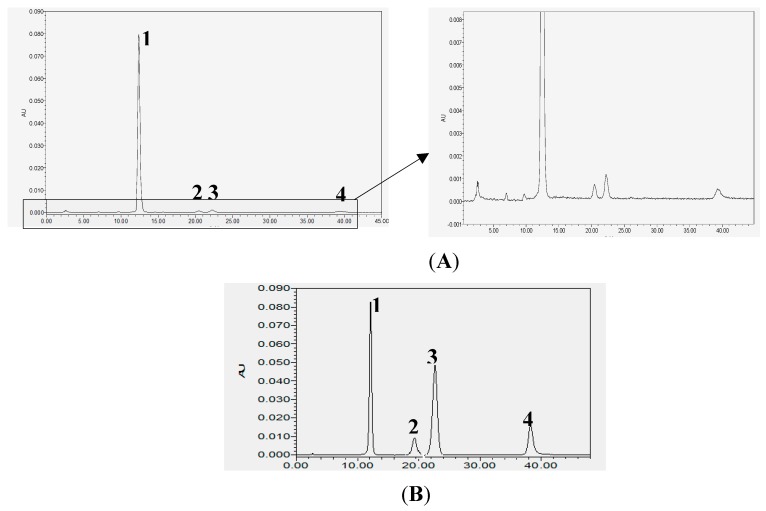
Representative HPLC chromatogram acquired at 270 nm of flavonoids from POE (**A**) and flavonoid standards (**B)**. Peaks were tentatively identified as (**1**) rutin, (**2**) hesperidin, (**3**) myricetin, (**4**) quercetin.

In order to reveal the approximately biological active components in POE, total flavonoids from *Portulaca oleracea* was detected by using high performance liquid chromatography (HPLC). The chromatogram of chemical compositions in POE was shown in Figure 1A. From the elution order, the main peaks at Rt = 12.455, 20.148, 22.210 and 39.226 were flavonoids. The chemical constituents and content were measured by rutin, hesperidin, myricetin and quercetin as reference substance, and these four components in POE were verified after comparison with the respective standards. The content of four represented flavonoids in POE reached 51.4% (0.5 g flavonoids per gram of POE extract). Their chemical structures were presented in Figure 1B, which were identified in previous study [12,13].

### 2.2. Effects of POE on Serum Biochemical Indicator in Alcoholic Liver Injury Rats

As shown in Table 1, compared with the normal control (NC) group, the serum level of alanine aminotransferase (ALT), aspartate aminotransferase (AST), alkaline phosphatase (ALP) and triglyceride (TG) significantly elevated (*p* < 0.01) in alcoholic liver injury model (ALI) group. The level of ALT, AST, ALP and TG was dose-dependently reduced in the low, medium, high dose of *Portulaca Oleracea* extract (POE-L, POE-M, POE-H, 25, 50, 100 mg/kg) group and the bifendate (BF, 3.75 mg/kg) group compared with the ALI group (*p* < 0.05). The results suggested that POE could ameliorate the ethanol-induced liver injury in rats.

### 2.3. Effects of POE on Oxidative Damage Index in the Liver

**Figure 2:**
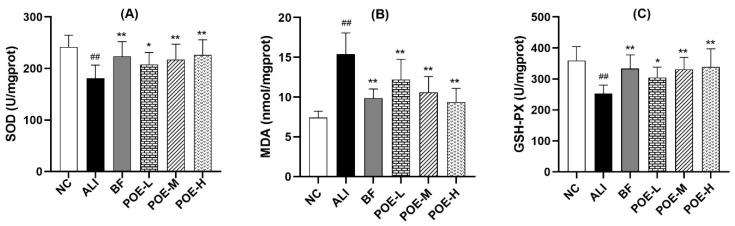
Effects of POE on oxidative damage index in alcoholic liver injury rats. (**A**) SOD activity, (**B**) MDA content, (**C**) GSH-PX activity. Values are presented as Mean ± SEM, *n* = 10. ^##^ *p* < 0.01 compared with NC group. * *p* < 0.05 and ** *p* < 0.01 compared with ALI group.

To investigate the intervention mechanisms of POE in hepatic injury induced by ethanol, superoxide dismutase (SOD) and malondialdehyde (MDA) and glutathione peroxidase (GSH-PX) were measured as parameters to evaluate the level of oxidative stress in liver. As shown in Figure 2, there were significant differences in the values of SOD, MDA and GSH-PX between the ALI group and the normal control group (*p* < 0.01).

The activity of SOD in liver tissue with low, medium and high dose of POE (25, 50, 100 mg/kg) group and BF (3.75 mg/kg) group elevated significantly (*p* < 0.01), when compared with the ethanol-induced ALI group. The content of MDA of low, medium and high dose of POE (25, 50, 100 mg/kg) group and BF (3.75 mg/kg) group decreased (*p* < 0.01), while the activity of GSH-PX in liver tissue increased significantly (*p* < 0.05 or *p* < 0.01) compared with the ALI group. The results indicate that POE exhibited protective effects on the oxidative damage of liver tissue.

### 2.4. Effects of POE on Inflammatory Cytokine and Mediators in Alcoholic Liver Injury Rats

**Figure 3:**
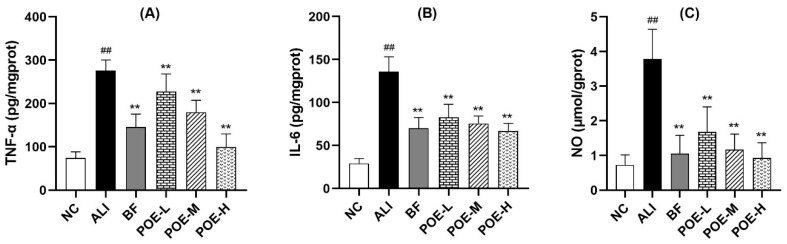
Effects of POE on inflammatory cytokine and mediators in alcoholic liver injury rats. (**A**) TNF-α, (**B**) IL-6, (**C**) NO. Values are presented as Mean ± SEM, *n* = 10. ^##^ *p* < 0.01 compared with NC group. ** *p* < 0.01 compared with ALI group.

As shown in Figure 3, the content of tumor necrosis factor-α (TNF-α), interleukin-6 (IL-6) and nitric oxide (NO) in ethanol-induced ALI group was markedly elevated, when compared to the normal control group. TNF-a, IL-6 and NO content in liver were significantly different in the POE low, medium and high dose groups (25, 50, 100 mg/kg) and ethanol-induced ALI group (*p* < 0.01). The results indicate that POE could improve the inflammation level of liver injury.

### 2.5. Effects of POE on Expression of miR-122

**Figure 4:**
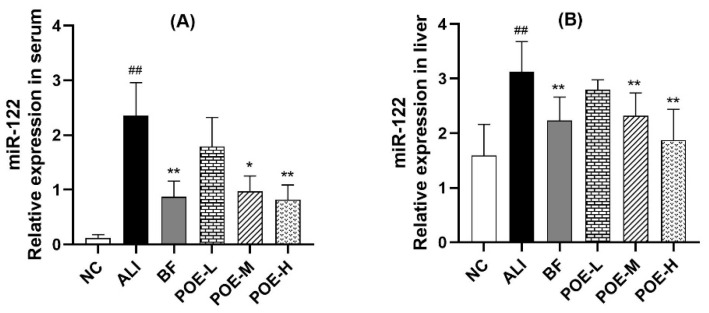
Effects of POE on expression of miR-122 in serum and liver of rats with alcoholic liver injury. (**A**) miR-122 in serum, (**B**) miR-122 in liver. Values are presented as Mean ± SEM, *n* = 6. ^##^ *p* < 0.01 compared with NC group. * *p* < 0.05 and ** *p* < 0.01 compared with ALI group.

As shown in Figure 4, the relative expression of miR-122 in serum and liver in the ALI group increased significantly (*p* < 0.01), when compared with the NC group. The expression of miR-122 in serum and liver at medium and high dose of POE groups (50, 100 mg/kg) significantly decreased (*p* < 0.05 or *p* < 0.01) compared with ALI group. These results suggested that POE could reduce the expression of miR-122 in serum and liver of rats with alcoholic hepatitis.

### 2.6. Effects of POE on the mRNA Expression of ACC1 and LPL

**Figure 5:**
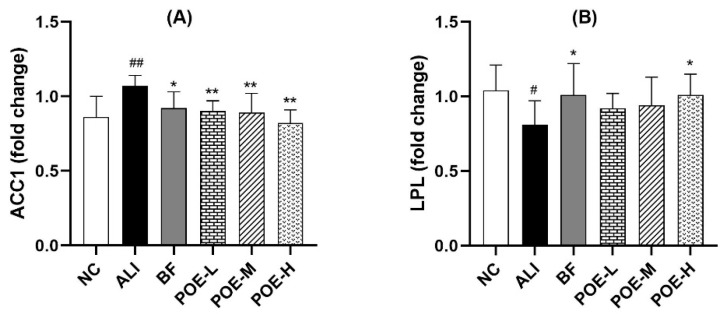
Effects of POE on the mRNA expression of ACC1 and LPL in liver of rats with alcoholic liver injury. (A) the mRNA expression of ACC1, (B) the mRNA expression of LPL. Values are presented as Mean ± SEM, *n* = 6. ^#^ *p* < 0.05 and ^##^ *p* < 0.01 vs. NC group. * *p* < 0.05 and ** *p* < 0.01 vs. ALI group.

As shown in Figure 5, the results showed that the mRNA expression of acetyl coenzyme A carboxylase (ACC) 1 and lipoprotein lipase (LPL) in the liver in ALI group increased (*p* < 0.01 or *p* < 0.05) compared with the NC group. The mRNA expression of ACC1 significantly decreased in the liver tissue at POE low, medium and high dose groups (25, 50, 100 mg/kg) (*p* < 0.01), when compared with the ALI group. The mRNA expression of LPL increased in the liver tissue of high dose of POE group (100 mg/kg) and BF group (*p* < 0.05).

### 2.7. Effects of POE on the Protein Expression of ACC1 and LPL

**Figure 6:**
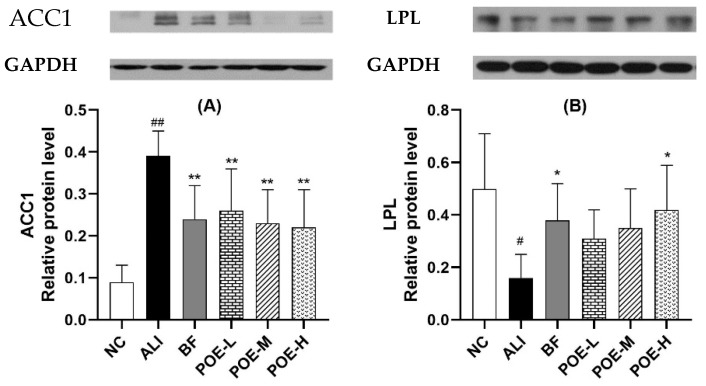
Effects of POE on the protein expression of ACC1 and LPL in liver of rats with alcoholic liver injury. (**A**) the protein expression of ACC1, (**B**) the protein expression of LPL. Values are presented as Mean ± SEM, *n* = 6. ^#^ *p* < 0.05 and ^##^ *p* < 0.01 vs. NC group. * *p* < 0.05 and ** *p* < 0.01 vs. ALI group.

ACC1 and LPL protein expression was studied using the Western blotting analysis. As shown in Figure 6, the expression level of ACC1 protein in the liver of the model group was down-regulated in the POE groups (25, 50, 100 mg/kg) as compared to the ALI group (*p* < 0.01). The level of LPL protein showed a pronounced difference when treated with POE at high dose, compared with ALI group (*p* < 0.05).

## 3. Discussion

*Portulaca oleracea* is a kind of food and medicine homologous product. Previous studies have showed that it has many pharmacological effects and is widely used against liver injury [31], which is more suitable for the development of clinical hepatoprotective drugs. The objective of this study was to investigate the main active components and potential protection mechanisms of *Portulaca oleracea* on acute alcoholic liver injury. In our study, HPLC was used to detecte the chemical constituents and content by rutin, hesperidin, myricetin and quercetin as reference substance, and the content of four represented flavonoids in POE reached 51.4%. Four flavonoids including rutin hesperidin, myricetin and quercetin in POE were identified. Numerous studies have shown that rutin and quercetin have a wide range of pharmacological effects and have hepatoprotective effects in liver injury models, such as acetaminophen-induced, CCl_4_-intoxicated liver injury and non-alcoholic fatty liver disease [22,37,38,39]. The identification of chemical constituents in POE provided a basis in the further study of pharmacological effects of POE.

The liver is the main organ of alcohol metabolism. Excessive drinking can lead to different degrees of alcoholic liver injury, which is reversible during early stages of liver injury. In this study, our liver injury model, which was induced by ethanol, is the usually used ALD animal model for evaluating the hepatoprotective effects of drugs [5,40,41,42]. Currently, liver injury can be evaluated by monitoring serum biomarkers such as ALT, AST and ALP [43]. In this study, POE significantly reduced the increase of blood biochemical parameters such as ALT, AST and ALP, and mitigated the ethanol-induced liver damage. Another important mark of alcoholic liver injury is showed by the elevated content of serum TG, which is a sign of steatosis of the liver [9]. Excessive intake of ethanol would cause obstacles in the circulation of tricarboxylic acid and the oxidation of fatty acids, which could affect the fat metabolism and lead to a large amount of TG accumulation in the liver, and then increase the content of TG in the blood. In our study, treatment with POE obviously decreased the TG content in serum, which indicated that POE could obviously improve the hepatocyte steatosis. 

The liver has important antioxidant enzymes, such as SOD, GSH-PX, GST etc. Excessive alcohol intake reduces the activity of antioxidant enzymes in the liver, destroys the oxidation–antioxidant balance of the body, thereby causing oxidative stress which further leads to a chain reaction of lipid peroxidation, mitochondrial function damage, endoplasmic reticulum stress and immune inflammation caused by liver damage [44]. MDA is generally considered to be an important marker of lipid peroxidation [35]. Inflammation has a pivotal role in the alcohol-induced liver injury as the source of oxidative injury [45]. TNF-α and IL-6 are the two major inflammatory cytokines found in the alcohol-induced liver injury. Alcohol can stimulate the activation of upstream nuclear transcription factor-κB (NF-κB) pathway in liver macrophages, which further induces the expression of the two cytokines [46,47]. Excessive NO, being an inflammatory mediator, can contribute to the liver injury [35,48]. After POE treatment, the activity of SOD and GSH-PX in liver was significantly elevated and the content of MDA, TNF-α, IL-6 and NO was decreased. Therefore, POE had antioxidant capacity, and could inhibit the alcohol-induced inflammatory reaction.

MicroRNAs (miRNAs) are a class of small noncoding RNAs of 18–25 nucleotides in length, which can play important regulatory role in animals and plants by targeting mRNAs for cleavage or translational repression [49,50]. Circulating miRNAs are present in the serum and plasma of humans and other animals in a remarkably stable manner and can serve as potential biomarkers for the detection of various cancers and other diseases [51]. MiRNAs can be detected in serum or plasma in liver disease such as drug-induced liver injury and alcoholic hepatitis [52,53]. MiR-122 is a potential biomarker for the diagnosis of alcoholic liver injury [54,55]. Therefore, we detected the relative expression of miR-122 in serum and liver tissues respectively. The results showed that the relative expression of miR-122 in serum and liver in the ALI group increased significantly. As well, the serum expression of miR-122 is highly up-regulated, however in the liver, the expression of miR-122 is only slightly but significantly higher in ALI group compared to NC rats. The likely reason for this is the relationship between the source of circulating miRNAs in blood and the cells of diseased tissues remains unclear at present. It is reported that the expression profile of serum miRNAs was consistent with that of blood cells in healthy subjects (Pearson R = 0.9206). However, in lung cancer and other diseases, the consistency between serum and blood cells decreased significantly (Pearson R = 0.4492). It suggested that that under normal conditions most serum miRNAs are derived from circulating blood cells. However, in disease states some miRNAs in the serum may come from diseased tissue cells [51].

It was found that miR-122 silencing could improve the liver steatosis and regulate the hepatic lipid metabolism, which resulted in reduced plasma cholesterol levels, increased hepatic fatty-acid oxidation, and a decrease in hepatic fatty-acid and cholesterol synthesis rate. Furthermore, numerous key genes that regulated lipid metabolism were also inhibited, such as FASN, ACC and so on, due to the miR-122 silencing [9,10]. LPL is the key enzyme of lipid metabolism, which is positively correlated with the miR-122 expression in chronic hepatitis [56]. LPL plays an important role in the process of lipid metabolism and transport, which is primarily synthesized in the adipose tissue, heart and skeletal muscle. The main function of LPL is to hydrolyze the core TG in the TG-rich lipoproteins, like the very low density lipoprotein (VLDL) from the liver and the chylomicron from the intestine yielding glycerol and free fatty acids (FFAs) for uptake by tissues, with a resulting decrease in plasma TG levels [57,58,59]. Acetyl coenzyme A carboxylase (ACC) is a rate-limiting enzyme that catalyzes the first step of fatty acid synthesis and metabolism and plays an important role in the lipid metabolism. ACC1 is mainly expressed in liver and catalyzes the synthesis of long-chain fatty acids, thereby promoting the progression of hepatic steatosis [60,61,62]. In our study, POE prevented the ethanol-induced liver injury by decreasing the expression of miR-122, ACC1 mRNA and protein, and increasing the expression of LPL-mRNA and protein in rats. These results indicated that POE could improve the lipid metabolism disorder caused by alcoholic liver injury.

In summary, this study demonstrated that POE exhibited protective effects on acute alcoholic liver injury in rats. Studies on the oxidative damage and inflammatory cytokines, together with qRT-PCR and Western blot experiments revealed that POE could regulate the expression of the vital liver biomarker miR-122, ACC1 and LPL in the lipid metabolism. POE stands a promising treatment of liver injury and may be further developed and utilized in the clinical scenario.

## 4. Materials and Methods

### 4.1. Materials

The *Portulaca oleracea* extract (No. ZL20150629) were obtained from Nanjing Zelang Medical Technology Co., Ltd. (Nanjing, China). The basic process was that the crude drugs of *Portulaca oleracea* was macerated with 75% aqueous ethanol for 2 h two times. The combined extract were concentrated under reduced pressure and sprayed drying and the powder was obtained. The product specification was 1 g extract/10 g crude drug of *Portulaca oleracea*. The *Portulaca oleracea* extract (POE) powder were weighed and dissolved with distilled water and diluted to 10 mg/mL for animal administration. The chemical compositions and content of POE were determined by HPLC. Waters Alliance 1525 Separation module equipped with binary pumps, a manual injector, a Waters 2998 variable wavelength scanning UV detector, and a 250 mm × 4.6 mm × 5 μm syncronis C18 column was used. HPLC analysis conditions: Methanol (A)-acetic acid water ((B), 1%), 0–45 min (A: B = 0.45:0.55); flow velocity: 1 mL/min, 2 μL injection; wavelength of detection: 270 nm; column temperature: 30 °C.

### 4.2. Chemicals and Reagents

Rutin, hesperidin, myricetin and quercetin (98% HPLC grade purity) were purchased from Chengdu Alfa Biotechnology Co., Ltd. (Chengdu, China). HPLC grade methanol and glacial acetic acid were obtained from Fuchen (Tianjin) Chemical Reagent Co. Ltd. (Tianjin, China). Anhydrous ethanol (analytical pure AR) were obtained from Tianjin Hengxing Chemical Reagent Manufacturing Co., Ltd. (Tianjin, China).

The assay kits for alanine aminotransferase (ALT), aspartate aminotransferase (AST), alkaline phosphatase (ALP) and triglyceride (TG) were procured from Shanghai Fuxing Long March Medical Science Co., Ltd. (Shanghai, China). Superoxide dismutase (SOD) and malondialdehyde (MDA), glutathione peroxidase (GSH-PX) and nitric oxide (NO) kits were obtained from Nanjing Jian Cheng Institute of Biological Engineering (Nanjing, China). Tumour necrosis factor-α (TNF-α) and interleukin-6 Enzyme-Linked Immuno Sorbent Assay (ELISA) kits were purchased from Suzhou Calvin Biotechnology Co., Ltd. (Suzhou, China).

Moloney Murine Leukemia Virus (M-MLV) Reverse Transcriptase, SYBR Premix Ex Taq II and DL2000 DNA Marker were supplied by TaKaRa (Dalian, China). Primary antibodies of ACC1 and LPL were purchased from Abcam (Cambridge, Britain). Antibody of GAPDH and HRP-conjugated goat anti rabbit IgG and goat anti mice were procured from Tiandeyue (Beijing) Biotechnology Co., Ltd. (Beijing, China).

### 4.3. Animals and Treatments

A total of 60 male specific pathogen free (SPF) degree male Wistar rats (200 ± 20 g) were obtained from the Jinan Pengyue Laboratory Animal Breeding Co., Ltd., Shandong, China (license approval number: SYXK (Lu) 20140007). The rats were fed in SPF room of Experimental Animal Center of Henan University of Chinese Medicine with food and water *ad libitum*. The animals were acclimatized for 7 days prior to the experiments. Temperature and relative humidity were respectively regulated at 20 ± 2 °C and 40–60%, with a cycle of 12 h each light/dark. The animal experiments were approved by the Animal Care and Use Committee of Henan University of Chinese Medicine.

The rats were randomly divided into 6 groups: normal control (NC) group, group (ALI), low, medium and high dose of POE (25, 50, 100 mg/kg, equivalent to 5 times, 10 times and 20 times of adult dosage respectively) groups, and bifendate (BF, 3.75 mg/kg, equivalent to 10 times of adult dosage in a day) group, with consecutive oral gavage for 7 days. POE groups were administrated corresponding drugs by intragastrical administration. NC and ALI groups received the same volume of distilled water. The ALI models in ALI, POE and BF groups were established on the 1st day after dose according to the references. Except for the normal control group, the other 5 groups were given 50% ethanol at 8 mL/kg by gavage 2 h later with repeated administration after 6 h, for 7 days.

The rats in each group were intraperitoneally injected with 10% chloral hydrate after 12 h of the last intragastric administration of ethanol. Blood was collected from abdominal aorta and serum was separated by centrifugation at 5000 rpm for 15 min at 4 °C. Serum samples (200 μL) were used for blood biochemical analysis and the remaining serum was frozen at −80 °C for reserve. The liver was immediately removed from each animal postmortem and divided into small pieces (100 mg) and frozen at –80 °C until analysis. One piece of the liver was made into 10% homogenate for oxidative stress and inflammation detection. And the remaining liver was used for detecting the expression of miR-122 and protein.

### 4.4. Blood Biochemical Analysis

The levels of serum ALT, AST, ALP and TG were measured by AU400 automatic biochemical analyzer (OLYMPUS Co., Ltd., Tokyo, Japan).

### 4.5. Liver Oxidative Stress and Inflammation Detection

The activity of SOD in the liver homogenate was detected by the microplate assay, the content of MDA and NO and the activity of GSH-PX were detected by colorimetry, and the content of inflammatory factors, TNF-α and IL-6 in liver tissue were detected by ELISA.

### 4.6. Real-Time Fluorescence Quantitative polymerase chain reaction (PCR) of MiR-122 in Serum and Liver

Total RNA was extracted from 200 µL of serum and 100 mg of liver respectively using the TRIZOL reagent according to kit instructions. The purity of isolated RNA was determined by OD 260/280 using a NanoDrop-2000 (Thermo Scientific, Worcester, MA, USA), with the value around 2.0 indicating high purity.

Expression of miR-122 was performed by using the Reverse Transcriptase M-MLV miRNA Detection Kit and SYBR Premix Ex TaqII. The mixture was incubated at 16 °C for 30 min, 42 °C for 40 min and 85 °C for 5 min to generate a library of miRNA cDNAs.

Real-time PCR reaction system included cDNA, SYBR Premix PCR Master Mix and miR-122 specific RT primers. The PCR assays were performed with an ABI 7500 fluorogenic quantitative PCR instrument (Applied Biosystems, Foster, CA, USA) in a 96-well reaction plate. The PCR reaction conditions were as follows: pre-denaturation at 95 for 30 s, and 40 cycles of 95 °C for 5 sec and 60 °C for 40 s. The specific primers were synthesized by Invitrogen (Carlsbad, CA, USA). The sequence of miR-122 sense primer: 5′-TGGAGTGTGACAATGGTG-3′, and antisense primer: 5′-GTGCGTGTCGTGGAGTCG-3′.

Melting curve analysis was performed to validate the specificity of the expected PCR product. The cycle threshold (Ct) was defined as the number of cycles required for the fluorescent signal to reach the threshold in PCR. MiR-39 was used as internal reference gene to normalize gene expression. The formula 2 − ΔΔCT was used to calculate the levels of miRNAs in serum and liver, where ΔΔCt = (CtmiRNA − CtmiR-39) treated − (CtmiRNA − CtmiR-39) untreated.

### 4.7. Quantitative Real-Time PCR (qRT-PCR) of ACC and LPL mRNA in Liver

The extracted total RNA of liver was used for reverse transcription of cDNA. The cDNA was synthesized by using M-MLV first chain synthesis kit (Invitrogen, Carlsbad, CA, USA). qPCR was measured using a SYBR green PCR kit (Thermo Fisher Scientific Inc., Waltham, MA, USA) in ABI PCR instrument. The amplification reaction conditions were as follows: 94 °C for 2 min; 40 cycles at 94 °C for 30 s, 56 °C for 30 s, and 72 °C for 30 s. The specific primers were synthesized by Sangon Biotech Co., Ltd. (Shanghai, China). The sequence of ACC1 sense primer was: 5′-TACCTCAATCTCAGCATAGC-3′, and antisense primer: 5′-AGCAGTTACACCACATACAT-3′. The sequence of LPL sense primer: 5′-CCAGCTGGGCCTAACTTTGA-3′, and antisense primer: 5′-CCAGCTGGGCCTAACTTTGA-3′. GAPDH was used as internal reference gene.

### 4.8. Western Blot of ACC and LPL protein in Liver

The liver tissues of the rats were homogenized in the radio immunoprecipitation assay (RIPA) protein extraction kit (Tiandeyue Biotechnology Co., Ltd., Beijing, China) containing 1 mmol/L phenylmethylsulfonyl fluoride (PMSF, Amresco, Boise, ID, USA) and 1% protease inhibitors (Roche, Basel, Swit). The concentration of protein was determined by using a BCA kit (Tiandeyue, Beijing, China). The protein was separated by sodium dodecyl sulfate-polyacrylamide gel electrophoresis (SDS-PAGE) and transferred to a polyvinylidene difluoride (PVDF) membrane. The membrane was blocked in 5% BSA-TBST for 30 min and incubated with the primary antibodies (ACC1, 1:2000; LPL, 1:500) overnight at 4 °C. 

After washing with TBST 5 times, the membranes were incubated with HRP-conjugated secondary anti-rabbit or anti-mouse antibodies (1:10,000) for 40 min at room temperature. The protein bands were visualized using ECL reagent (Merck Millipore, Darmstadt, Germany)) and the integral optical density (IOD) of the bands was analyzed by TotalLab Quant.

### 4.9. Statistical Analysis

The data was represented as Mean ± SEM. Statistical differences were analyzed by the One-way analysis of variance (ANOVA) followed by Dunnett’s post hoc test. A value of *p* < 0.05 was considered to statistically significant.

## 5. Conclusions

Our study showed that POE exhibited hepatoprotective effects in the acute alcoholic liver injury in rats, and its mechanism might be related to the inhibition of lipid peroxidation and oxidative stress, reduction in the excessive release of inflammation cytokine, and by alleviating the inflammatory reactions. Furthermore, POE alleviated liver injury through regulating the miR-122, lipid metabolism-related genes and protein ACC1 and LPL. Thus, our study provided an experimental basis for the safe use in the clinical scenario and laid the foundation for the development and utilization of *Portulaca oleracea* as hepatoprotective herb.

## Figures and Tables

**Table 1:** Effects of POE on serum biochemical indicator in alcoholic liver injury rats.

Groups	Dose (mg/kg)	ALT (U/L)	AST (U/L)	ALP (U/L)	TG (mmol/L)
NC	-	25.65 ± 4.80	88.60 ± 13.41	142.35 ± 37.21	0.56 ± 0.22
ALI	-	42.38 ± 8.01 ^##^	137.20 ± 26.91 ^##^	254.71 ± 53.55 ^##^	1.00 ± 0.31 ^##^
BF	3.75	31.26 ± 6.04 **	110.60 ± 18.06 **	177.05 ± 43.79 **	0.77 ± 0.28 *
POE-L	25	34.87 ± 6.10 *	117.10 ± 13.91 *	209.00 ± 55.79 *	0.75 ± 0.23 *
POE-M	50	31.37 ± 6.92 **	113.80 ± 16.49 **	191.91 ± 56.88 **	0.70 ± 0.20 *
POE-H	100	30.93 ± 6.23 **	109.44 ± 14.68 **	185.90 ± 47.86 **	0.59 ± 0.23 **

Data are expressed as the mean ± Standard Error of Mean (SEM), *n* = 10. ^##^ *p* < 0.01 vs. NC group. * *p* < 0.05, ** *p* < 0.01 vs. ALI group.
